# Chemistry of Medicinal Plants, Foods, and Natural Products

**DOI:** 10.1155/2014/252516

**Published:** 2014-06-04

**Authors:** Shixin Deng, Shao-Nong Chen, Jian Yang

**Affiliations:** ^1^Research and Development Department, Morinda Inc., American Fork, UT 84003, USA; ^2^UIC/NIH Center for Botanical Dietary Supplements Research, Department of Medicinal Chemistry and Pharmacognosy, College of Pharmacy, University of Illinois, Chicago, IL 60612, USA; ^3^Western Pacific Tropical Research Center, College of Natural and Applied Sciences, University of Guam, Mangilao, GU 96923, USA

Medicinal foods and plants have been widely used as foods, dietary supplements, or medicines worldwide and demonstrated a diversified health benefits with a long history. The raw materials and finished products of botanicals are becoming increasingly popular to public and scientific communities, highlighting the need for analytical methodology to ensure the quality. The safety and efficacy of these medicinal natural products are closely associated to their identify, authenticity and quality, which in turn relate to many factors, such as geographical conditions (soil, sunlight, precipitation, and air) and post-growth factors (harvesting, storage, transportation, manufacturing processes, etc.). As such, it is always a challenge for scientific researchers to evaluate safety and efficacy of the complicated plant matrix. In this special, we have invited 15 original research articles addressing the novel analytical method development and validation, methodology and instrumentation improvement, chemical characterization, biological activities of plant materials, extracts, and pure phytochemicals.

The first paper of this special issue investigates the feasibility of electronic tongue and multivariate analysis for discriminating the specific geographical origins of a Chinese green tea with protected designation of origin, and the authors concludes that electronic tongue and chemometrics can provide a rapid and reliable tool for discriminating the specific producing areas of Longjing. The second paper describes a quantitative method of flavonoids and chlorogenic acid in the leaves of* Arbutus unedo* L. by using HPTLC and their antioxidant activities by DPPH. The third paper reports a reliable HPLC method for the determination of aflatoxin M1 in eggs. The forth paper explores the characteristics of the anthocyanin and flavonol composition and content in grapes from plants resulting from intraspecific crosses of* Vitis vinifera* varieties Monastrell × Cabernet Sauvignon, Monastrell × Syrah, and Monastrell × Barbera. The fifth paper presents a rapid and sensitive method for determining aesculin of* Cortex fraxini* in rat by using HPLC-MS/MS with QTOF as a detector.

The sixth paper reports antioxidant activity and free radical scavenging capacity of the essential oil and three different extracts of wildly grown* Mentha longifolia*. Meanwhile, the study also establishes volatile and phenolic profiles of essential oil and different extracts of wild mint (*Mentha longifolia*) from the Pakistani Flora by using GC-MS method. The seventh paper establishes a phytochemical profile of* Cornus mas* by identifying the biological constituents in the fruits with the advanced UPLC-MS-TOF technology. Additionally, it reports DNA protective and antigenotoxic activities of the main phytochemicals. The eighth paper proposes a GC-MS fingerprint method for comparison of volatile components in the different plant parts (leaves and roots) of* Agrimonia eupatoria*. The ninth paper describes the isolation of *α*- and *β*-carotene from crude palm oil and evaluates their antioxidant potential in an in vitro model. The tenth paper focuses on the development of a sensitive and specific gas chromatographic-mass spectrometry with selected ion monitoring (GC-MS/SIM) method for simultaneous identification and quantification of *α*-asarone, *β*-asarone, and methyl eugenol of* Acorus tatarinowii* Schott in rat plasma.

The eleventh paper addresses in vivo evaluation of the antioxidant activity of deacetylasperulosidic acid (DAA), a major iridoid in* Morinda citrifolia* (noni) fruit, and the study concludes that DAA contributes to the antioxidant activity of noni juice by increasing superoxide dismutase activity. The twelfth paper demonstrates that 1H NMR spectroscopy is a suitable detection technique in the analysis of various phytosterol forms in natural extracts. The thirteenth paper investigates the effects of varying temperatures with constant pressure of solvent on extraction efficiency of two chemically different alkaloids, camptothecin (CPT) from stem of* Nothapodytes nimmoniana* (Grah.) Mabb. and piperine from the fruits of* Piper nigrum* L., and determines a compound specific extraction for the two target alkaloids. The fourteenth paper isolates and purifies a protein called agathi leaf protein (ALP) from* Sesbania grandiflora* Linn. (agathi) leaves and evaluates its antioxidant, antibacterial, and cytoprotective activity. The fifteenth paper describes the characterization of nine polyphenols in Korean Prostrate Spurge (*Euphorbia supina*) by using HPLC-MS/MS, and the dose-dependent antioxidant activities of flavonoids were also observed.

*Shixin Deng* *Shixin Deng* *Shao-Nong Chen* *Shao-Nong Chen* *Jian Yang* *Jian Yang*

